# Covert Reorganization of Implicit Task Representations by Slow Wave Sleep

**DOI:** 10.1371/journal.pone.0005675

**Published:** 2009-05-25

**Authors:** Juliana Yordanova, Vasil Kolev, Ullrich Wagner, Rolf Verleger

**Affiliations:** 1 Department of Neurology, University of Lübeck, Lübeck, Germany; 2 Department of Neuroendocrinology, University of Lübeck, Lübeck, Germany; 3 Institute of Neurobiology, Bulgarian Academy of Sciences, Sofia, Bulgaria; 4 Department of Neuroscience, University Medical School, University of Geneva, Geneva, Switzerland; Victoria University of Wellington, New Zealand

## Abstract

**Background:**

There is evidence that slow wave sleep (SWS) promotes the consolidation of memories that are subserved by mediotemporal- and hippocampo-cortical neural networks. In contrast to implicit memories, explicit memories are accompanied by conscious (attentive and controlled) processing. Awareness at pre-sleep encoding has been recognized as critical for the off-line memory consolidation. The present study elucidated the role of task-dependent cortical activation guided by attentional control at pre-sleep encoding for the consolidation of hippocampus-dependent memories during sleep.

**Methodology:**

A task with a hidden regularity was used (Number Reduction Task, NRT), in which the responses that can be implicitly predicted by the hidden regularity activate hippocampo-cortical networks more strongly than responses that cannot be predicted. Task performance was evaluated before and after early-night sleep, rich in SWS, and late-night sleep, rich in rapid eye movement (REM) sleep. In implicit conditions, slow cortical potentials (SPs) were analyzed to reflect the amount of controlled processing and the localization of activated neural task representations.

**Principal Findings:**

During implicit learning before sleep, the amount of controlled processing did not differ between unpredictable and predictable responses, nor between early- and late-night sleep groups. A topographic re-distribution of SPs indicating a spatial reorganization occurred only after early, not after late sleep, and only for predictable responses. These SP changes correlated with the amount of SWS and were covert because off-line RT decrease did not differentiate response types or sleep groups.

**Conclusions:**

It is concluded that SWS promotes the neural reorganization of task representations that rely on the hippocampal system despite absence of conscious access to these representations.

**Significance:**

Original neurophysiologic evidence is provided for the role of SWS in the consolidation of memories encoded with hippocampo-cortical interaction before sleep. It is demonstrated that this SWS-mediated mechanism does not depend critically on explicitness at learning nor on the amount of controlled executive processing during pre-sleep encoding.

## Introduction

There is increasing evidence that sleep plays an important role for the plastic cerebral changes underlying memory consolidation [1]–[6]. At the neurobiological level of memory classification, a distinction has traditionally been made between a hippocampus-dependent memory system subserving explicit (declarative) memory formation, and a more heterogeneous hippocampus-independent system underlying different types of implicit (procedural) memory formation [6]–[11]. One central mechanism subserving memory consolidation during sleep involves the hippocampo-cortical interplay that is assumed to support the transfer of newly encoded memories from a temporary buffer in the hippocampus to a long-term store in the neocortex [7]–[8], [12]–[15]. This mechanism has been associated with explicit memory formation, and with a specific sleep stage, i.e., slow wave sleep (SWS) in both animals [16]–[17] and humans [8], [14], [18]–[19].

Explicit remembering in humans is accompanied by conscious access to memory contents, which allows attentive and controlled retrieval, whereas implicit memory retrieval remains partly or fully out of awareness [10]–[11], [20]. A similar distinction can be made for the initial acquisition (learning) phase of memory formation: Only during explicit learning are cortical activation patterns modified by conscious executive control systems, in contrast to implicit learning [6], [11]. Importantly, Robertson et al. (2004) [21] have demonstrated that improvement of performance in a serial reaction time task (SRTT) where explicit and implicit memory systems are activated in parallel occurs after sleep only when subjects are trained in explicit but not in implicit pre-sleep conditions. This emphasizes the key role of the explicit memory system for subsequent off-line consolidation. This also shows, however, that in addition to hippocampo-cortical activation linked to that system [8], task-dependent cortical activation guided by attentional (intentional) control at pre-sleep encoding may be critical for the subsequent consolidation of hippocampus-dependent memories during sleep.

**Figure 1:**
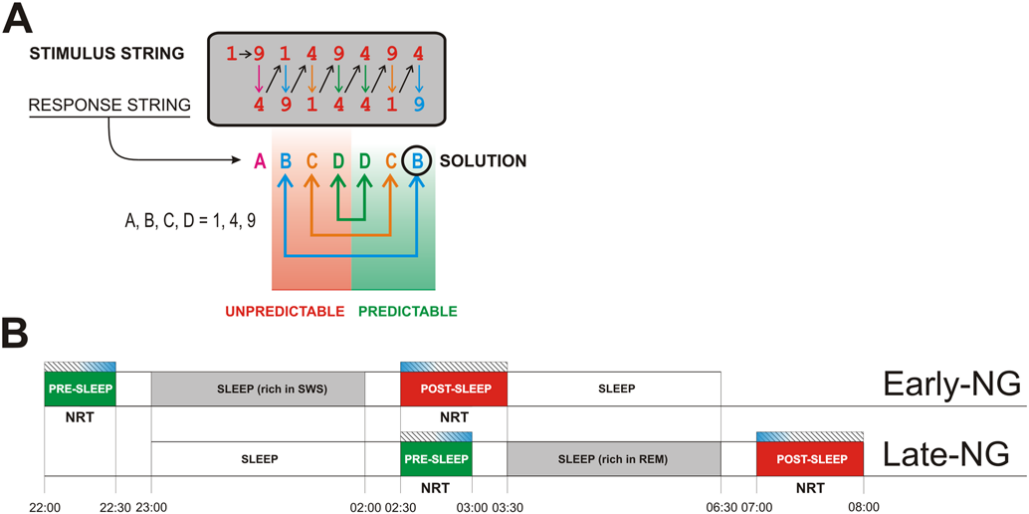
Experimental design of the study. (A) Schematic presentation of the paradigm. Black arrows present the consecutive steps in NRT task performance (e.g., the first two numbers 1 and 9 in the stimulus string lead to response 4 (A), then the same response (4) is compared with the next number from the stimulus string (1) leading to response 9 (B), and so on). Structure of the responses is given by letters showing the equal responses and in such a way forming the mirror structure (B, C, D - D, C, B). The final result is the last response (B) marked with SOLUTION which is followed by Enter. (B) The experimental protocol. NRT pre-sleep and post-sleep sessions are marked for the two sleep groups (Early-NG and Late-NG). Hatching bars present the time period of EEG recording. Blue shadings during EEG recordings present the time windows used to extract 35 artifact-free single sweeps for analysis.

The aim of the present study was to distinguish the roles of hippocampo-cortical engagement and attentionally controlled cortical activation for sleep-related memory consolidation, based on recent evidence that in certain memory tasks hippocampal processing is not strictly confined to explicit processes in memory formation and can therefore be dissociated from conscious control of task processing [22]–[23]. Specifically, we used here the Number Reduction Task (NRT) [24]–[25] that similar to the SRTT co-activates explicit and implicit systems [26]. In each trial of this task, consecutive responses to digit strings are produced to obtain a specific digit as the final result. Unknown to participants, the order of digits in the strings follows a regular pattern according to which the last three responses symmetrically mirror the preceding ones (Fig. 1A). Subjects can acquire both explicit and implicit knowledge of this hidden regularity. Explicit knowledge is acquired if subject become aware of the regularity (gain of insight), which allows them to qualitatively improve their task performance by reducing the number of responses in each trial [26]–[29]. Apart from this, also implicit knowledge of the hidden task regularity can be acquired: Even if the subjects are unaware of the hidden task structure, they implicitly speed up the responses that can be predicted relative to those that cannot be predicted by the regularity [26]–[27], [30]–[32]. Critically, functional neuroimaging studies have demonstrated that this implicit speeding is accompanied by enhanced activity of the medial-temporal lobe and the hippocampus to predictable relative to unpredictable responses [30]–[31]. Thus, the important property of the NRT employed here is that implicit learning of the relation between responses in this task activates strongly hippocampo-cortical networks [30]–[31] thus providing a condition in which involvement of the hippocampal system can be dissociated from processes of conscious attentional control in task performance [7].

In the present study, participants trained the NRT before sleep in order to create implicit neural representations of task material and performed a retest session after sleep, as reported in previous sleep studies with the NRT [26], [28]. Here, however, for the first time a direct monitoring of cortical activation patterns was made during task performance before and after sleep, using electroencephalographic recording (EEG). According to previous neuroimaging findings [30]–[31], pre-sleep involvement of hippocampo-cortical networks would be stronger for predictable than unpredictable responses. Thus, if the pre-sleep activation of hippocampal-cortical networks were the critical condition for sleep-dependent memory consolidation, a reorganization of neural task representations after sleep would only be expected for the implicitly processed predictable responses ( i.e., during processing of the second half of the digit string). Alternatively, sleep might support the reorganization of those task representations that received more attentional control at pre-sleep encoding (as observable in EEG patterns) regardless of predictability vs. unpredictability of responses in relation to the hidden task structure.

In the present study, these issues were addressed by analysis of slow EEG potentials. Slow potentials (SPs) appear as positive or negative DC shifts of the ongoing EEG during task processing and last up to several seconds [27], [33]–[34]. The magnitude of negative SPs reflects the amount of controlled processing, whereas the regional distribution of negative SPs reflects the localization of activated task representations [34]–[36]. Applied to the current sleep study, a difference in the level of attentional control of predictable and unpredictable response processing at encoding before sleep would be reflected by different amplitude of negative SPs. If sleep promoted an off-line reorganization of neural task representations depending on either hippocampo-cortical or conscious control activations, this neural re-structuring would be manifested by a spatial re-distribution of negative SPs after sleep, or by magnitude reduction due to facilitation of processing.

To distinguish the presumed particular role of SWS for hippocampus-dependent memory consolidation, the present study used a half-night design to compare the reorganization of task representations to unpredictable and predictable responses in the NRT across retention intervals spanning either the early part of nocturnal sleep rich in SWS, or late part of nocturnal sleep, rich in rapid eye movement (REM) sleep.

## Results

The same version of the NRT and samples were used as in the study of Yordanova et al. (2008) [26], which focused on behavioral parameters of explicit vs. implicit knowledge generation in this task. Subjects performed on two task sessions, a pre-sleep one (3 blocks) and a post-sleep retest session (10 blocks). There were two sleep groups. Subjects from the first group were retested after having slept for 3 h in the first half of the night following a pre-sleep NRT training in the evening (early-night group, Early-NG), and subjects from the second group were retested after having slept for 3 h in the second half of the night following a pre-sleep training performed in the middle of the night (late night group, Late-NG) – Fig. 1B. During pre-sleep training and post-sleep retest, subjects' performance was monitored on-line. In parallel, EEG activity was continuously recorded from 28 scalp electrodes. Brain electric signals were used to evaluate SP markers of unpredictable vs. predictable response processing in the NRT.

### Distribution of Sleep Stages

Sleep EEG was recorded at C3 and C4 electrodes and sleep stages were classified in 30-second epochs according to standard criteria [37]. Sleep recordings confirmed the differential distribution of SWS vs. REM sleep in the early and late halves of the night (Table 1). Subjects in the Early-NG had substantially more SWS than those in the Late-NG (F(1,47 = 24.8, p<0.001), and subjects in the Late-NG, conversely, had substantially more REM sleep than those in the Early-NG (F(1,47) = 80.8, p<0.001). The two groups did not differ in the proportions of other sleep stages (p≥0.2) – Table 1.

**Table 1:** Distribution of sleep stages in the early- vs. late-night group.

	Early-night group	Late-night group	Early-NG vs. Late-NG	
			F(1,47)	P
Wake (%)	3.2±1.9	0.5±0.3	1.7	0.19
S1 (%)	7.4±1.2	6.8±0.9	0.2	0.66
S2 (%)	57.0±2.9	61.8±1.7	1.9	0.20
SWS (%)	26.6±2.9	9.6±1.6	24.8	**<0.001**
REM (%)	5.5±1.1	21.3±1.4	80.8	**<0.001**
Total sleep time (min)	188.3±4.5	191.3±2.9	0.3	0.61

Means±SEM are indicated. Data refer to the sleep interval between initial practice and retesting. Statistical results are from one-way ANOVA comparing early- and late-night group. Significant P-values are in bold.

S1, sleep stage 1; S2, sleep stage 2; SWS, slow wave sleep; REM, rapid eye movement sleep.

### Behavioral Results

Ratings of subjective feelings of sleepiness, activation, tension, boredom, motivation, and concentration were obtained before and after each session of initial practice and retest. In line with the results reported in Ref. [26], the two experimental groups did not differ on the whole in these variables also in the present subsample, as indicated by non-significant main effects of early vs. late night (all p>0.2). However, subjects felt more sleepy and less activated, motivated and concentrated in task sessions performed in the middle of the night (i.e., initial training for late-night group, retest for early-night group) than in sessions performed in the evening (initial training for early-night group) or in the morning (retest for late-night group) (p<0.05, for respective night-half×session interactions). A much stronger effect independent of sleep was an activating effect of task performance itself, i.e., subjects felt less sleepy and more activated at the end as compared to the beginning of a task session (p<0.001).

Presence of explicit knowledge (insight) after sleep was determined from NRT task performance by an abrupt reaction time (RT) decrease and a short-cut in sequential responding (Fig. 1A) and was confirmed by answers in the post-experimental questionnaire. All subjects whose insight into the hidden structure had been identified automatically by the task program were also able to verbalize the critical explicit rule knowledge correctly in open questions (i.e. using their own words), and they were also able to give correct solutions to new digit strings within 2 s. Within the sample used, eight subjects from the Early-NG (32%) and 5 subjects from the Late-NG (21%) gained insight after sleep, revealing a similar rate of insight in the two sleep groups (χ^2^ (1, n = 48) = 0.6, p = 0.4). However, in the first block after sleep used for SP analysis, none of the subjects had still gained any explicit knowledge of the hidden task regularity.

**Figure 2:**
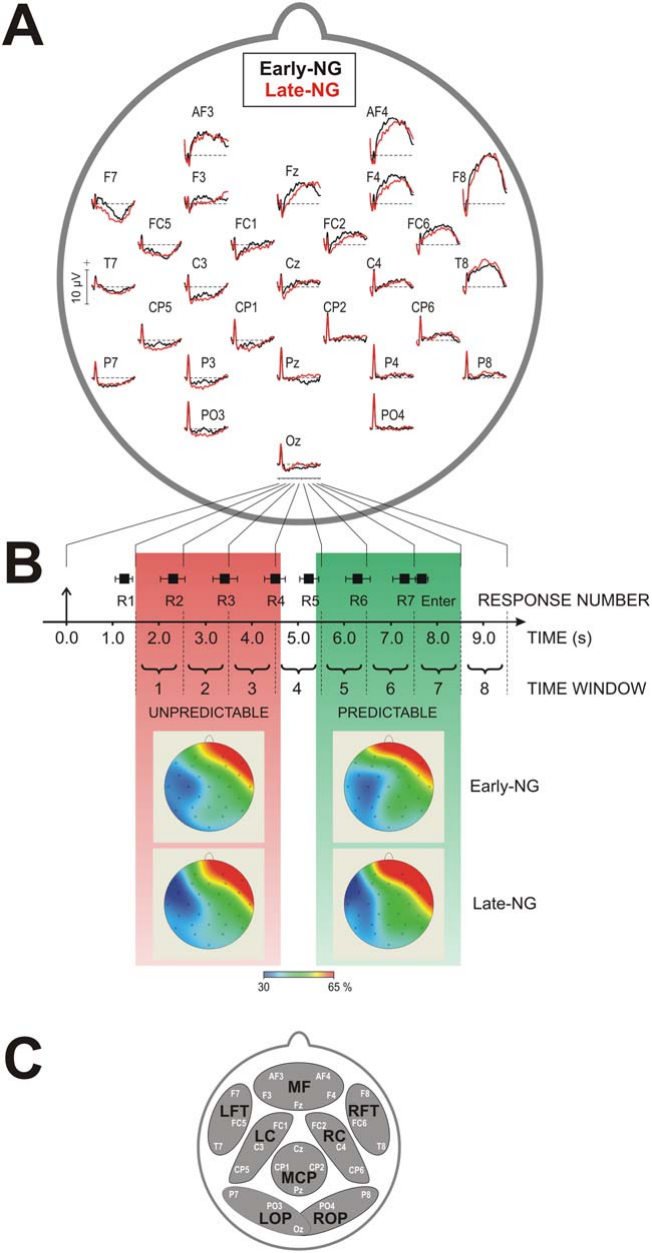
Measurements and analysis of slow potentials (SPs). (A) Grand average event-related slow potentials for the two sleep groups, Early-NG and Late-NG, for the pre-sleep period. (B) Mean reaction times and standard deviations for different responses (response numbers R1 to R7, and Enter) presented together with the 1-s time windows (1 to 8) used for SP measurements. SP measurement starts 1.5 s after string appearance in order to avoid the influence of the late ERP components. Response numbers are conditionally divided into two groups (response types) according to their position in the string and are labeled UNPREDICTABLE and PREDICTABLE. At the bottom, min-max transformed grand average maps for the two response types are shown. (C) Regions of interest used for SP analysis: MF - middle frontal, LFT - left fronto-temporal, RFT - right fronto-temporal, LC - left central, RC - right central, MCP - middle centro-parietal, LOP - left occipito-parietal, ROP - right occipito-parietal.

In the present experimental design, seven consecutive responses were produced (Response Number, R1–R7) – Fig. 2B. RTs were analyzed for each response number, with R2, R3, and R4 representing the unpredictable responses, and R5, R6 and R7 representing the predictable responses. To assess gain of implicit knowledge before sleep RTs were analyzed in the pre-sleep session. RT did not differ between the two sleep groups (F(1/46) = 1.95, p>0.15) but RTs to predictable responses were significantly shorter that RTs to unpredictable responses (F(1/46) = 12.24, p = 0.001).

**Figure 3:**
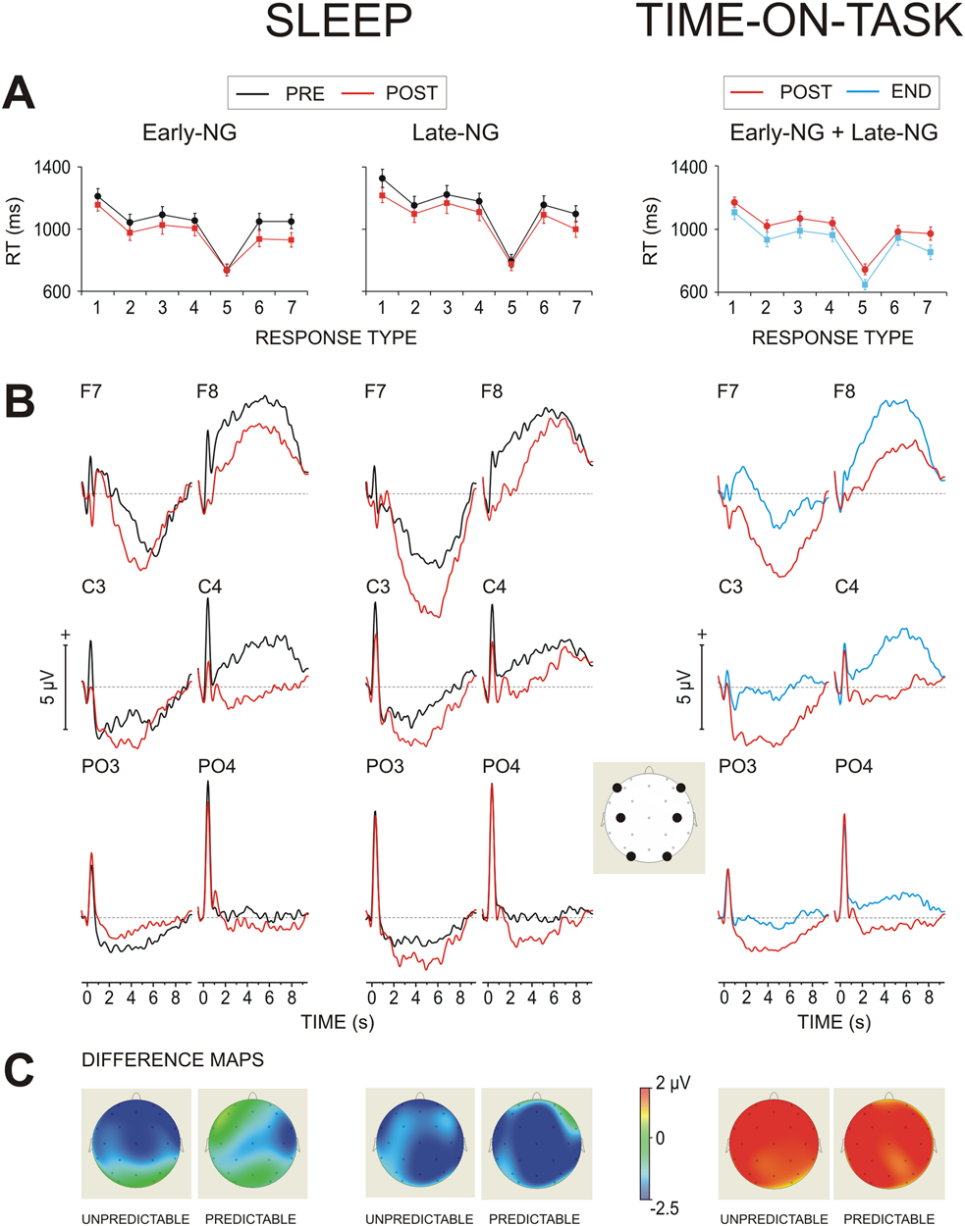
Effects of sleep and time-on-task on the reaction times (RTs) and slow potentials (SPs). (A) Group mean (±SE) RT values for the pre-sleep (PRE) and post-sleep (POST) period (left panel) and the effect of practice on the RTs (right panel) for early- and late-night groups pooled together. END - end of retest (task) period. (B) Grand average ERPs from six specific electrodes (with their locations shown in the empty map). Slow potentials are expressed after 1.5 s. (C) Difference maps (post-sleep minus pre-sleep) shown for the two response types, unpredictable and predictable (left panel), and difference maps of the end-of-retest minus beginning-of-retest period for both groups pooled together (right panel).

To assess effects of sleep RTs were subjected to a repeated measures analysis of variance with the between-subjects variable Sleep Group (Early-NG vs. Late-NG) and within-subjects variables Session (pre-sleep vs. post-sleep) and Response Number (R1–R7). As illustrated in Fig. 3A, RT decreased after sleep (F(1/46) = 25.2, p<0.001; mean RT before sleep 1077±28 ms vs. mean RT after sleep 1009±28 ms). This effect of Session did not depend on the sleep group (F(1/46) = 0.07, p>0.8, for the interaction), and was expressed for all seven responses (p<0.005 for simple test ANOVAs), with exception of R5 (F(1/46) = 0.22, p>0.6).

### Slow potentials

Slow potentials were obtained by averaging string-related EEG activity (Fig. 2A). Trials used for averaging contained 35 artifact-free sweeps from the last block of the pre-sleep session and the same number of artifact-free sweeps from the first block of the post-sleep session. For each subject, session, and electrode, mean values of slow potentials were measured for 8 consecutive 1-s time windows starting 1.5 s after string onset (Fig. 2A,B). Three parameters of SPs were evaluated: (1) Mean amplitudes, (2) Min-max normalized SPs, (3) Z-transformed SPs. Normalized SPs were used for analysis of spatial characteristics (see Materials and Methods).

SPs were analyzed for unpredictable and predictable response processing. As illustrated in Fig. 2B, quantification of unpredictable responses included time windows 1, 2 and 3 of the analysis epoch, whereas quantification of predictable responses included time windows 5, 6, and 7. Time window 4 was omitted from analysis because in this time window, both unpredictable and predictable response types could occur due to inter-trial and individual variations of RTs.

Figure 2C shows eight regions of interest (ROIs) formed for SP analysis: LFT - left fronto-temporal, LC - left pre-central, central and post-central (contralateral to the responding right hand), LOP - left occipito-parietal, RFT - right fronto-temporal, RC - right pre-central, central and post-central (ipsilateral to the responding right hand), ROP - right occipito-parietal, MF - mid-frontal, and MCP - mid-centro-parietal. The electrodes that were pooled together to form different ROIs are shown in Fig. 2C.

#### (A) Pre-sleep analysis

First, to rule out that any group in SP changes across sleep could be simply attributable to differences that were already present before the critical post-learning sleep period, and also to assess the amount of executive control in that period, we performed a comparison between the two groups only for the pre-sleep session. The results of this analysis are shown in Figure 2A,B. On the whole, SPs manifested a characteristic topographic pattern: Negative SPs were distributed primarily over the left hemisphere, whereas positive SPs appeared mainly over the anterior right hemisphere (ROI, F(7/322) = 58.5, p<0.001; left vs. right comparison, F(1/46) = 159.2, p<0.001). Negative SPs were mostly expressed over the left central and left frontal-temporal regions (Fig. 2B). Importantly, as illustrated in Fig. 2A, SP magnitude did not differ significantly between the Early-NG and Late-NG (F(1/46) = 0.7, p = 0.4). Also, the topography patterns were similar for the two groups (Fig. 2B). This was verified by the lack of significant interactions of Sleep Group with Topography (E or ROI): For all analyses of Z-transformed and min-max normalized SPs, F-values were <0.8, p>0.6.

Further, SP magnitude and regional distribution for unpredictable and predictable responses did not differ between the two sleep groups as indexed by the lack of significant interactions with the Response Type factor (p>0.3 for all Sleep Group×Response Type and Sleep Group×ROI or Electrode×Response Type interactions of original, Z-transformed, and min-max normalized SPs).

Before sleep, negative SPs at the left hemisphere did not differ significantly between unpredictable and predictable responses (F(2/92) = 0.12, p = 0.7), although at centro-posterior regions they tended to be larger for unpredictable responses, in contrast to the left temporo-frontal region (Response Type×ROI, F(2/92) = 6.5, p = 0.004). This effect was similar for the two sleep groups (Response Type×ROI×Sleep Group, F(2/92) = 0.33, p = 0.7).

#### (B) Across sleep analysis

The ANOVA design included the between-subjects factor Sleep Group (Early-NG vs. Late-NG) and the repeated-measures factors Session (pre-sleep vs. post-sleep), Response Type (unpredictable, in time windows 1, 2, 3 vs. predictable, in time windows 5, 6, 7), and ROI.

Figure 3B demonstrates changes of SPs between the two sessions, from before sleep to after sleep. SPs were overall more negative after sleep than before sleep (Session, F(1/46) = 6.27, p = 0.015). Importantly, SP negativization after sleep differentiated the two sleep groups. In the Late-NG, SPs were uniformly more negative after sleep than before sleep at all electrodes and for both response types (unpredictable and predictable) – Fig. 3B,C. In contrast, in the Early-NG, SP negativization after sleep depended on both region and response type (Fig. 3B,C): After early sleep (1) the SPs at occipito-parietal locations did not change significantly for any response type, and (2) the SPs at the left frontal-temporal and left central ROIs (LFT and LC) did not change for the second half of the string associated with the processing of predictable responses.

These differences between the Early-NG and Late-NG were verified statistically by analysis of difference SP values obtained by subtracting pre-sleep from post-sleep measures, which revealed a significant Sleep Group×ROI×Response Type (unpredictable vs. predictable) interaction (F(5/230) = 2.8, p<0.05). Testing simple effects of Sleep Group confirmed that group differences were present for both response types at the posterior left and right ROIs (LOP: F(1/46) = 9.7, p<0.01; ROP: F(1/46) = 4.5; p<0.05, Sleep Group×Response Type, p>0.4 for each of the LOP and ROP analysis). Yet, the Sleep Group×Response Type interactions were significant for the LFT (F(1/46) = 9.7, p<0.005) and LC (F(1/46) = 6.03, p<0.02) due to SP negativization for both the unpredictable and predictable responses in the Late-NG (Response type effect at LFT: F(1/22) = 0.01, p>0.9; at LC: F(1/22) = 0.28, p>0.6), and SP negativization only for unpredictable responses in the Early-NG (Response Type effect at LFT: F(1/24) = 50.6, p<0.001; at LC: F(1/24) = 20.2, p<0.001). No significant effects of Sleep Group were found for the other ROIs.

To characterize the changes of SP topography from the session before sleep to after sleep independently of individual variations in SP magnitude min-max normalized values of SPs were analyzed. Before sleep, the two sleep groups had similar relations among left ROIs (LFT, LC, LOP) in the course of string processing (Sleep Group×ROI and Sleep Group×ROI×Response Type, p>0.7). Yet, only after early sleep, did these relations change (Session×ROI×Response Type in the Early-NG: F(2/48) = 4.93, p = 0.01) due to the relative deactivation of the LOP ROI (effect of Session for this ROI in the Early-NG: F(1/24) = 3.2, p<0.05) and to differences between topography patterns of predictable and unpredictable responses in this sleep group, F(1/24) = 5. 35, p<0.05, see Supporting Information Text S1 and Fig. S1). The regional SP re-distribution only after early sleep was confirmed by z-transformed data (cf. Materials and Methods): A significant Session×Response Type×Electrode effect was found only in the Early-NG (F(27/648) = 2.1, p<0.05), but not in the Late-NG (F(27/594) = 0.72, p>0.7).

In separate analyses, the effects of explicit knowledge generation after sleep was tested for SP parameters by comparing groups of subsequent solvers with non-solvers. None of these analyses yielded significant effects of post-sleep insight on SP.

### Control analysis: Effects of Time-On-Task

Behavioral and SP parameters were compared between the first and last block of the retest session after sleep in order to provide a control reference framework for the changes in these parameters with procedural learning. Participants who gained insight had to be dropped from these analyses because these participants did not perform any more in the last block.

Figure 3A (right panel) demonstrates that RTs for all response numbers significantly decreased with time-on-task (F(1/33) = 14.9, p<0.001). In contrast to off-line learning across sleep, RT speeding across practice was found for each response number (Time-on-task×Response Number, F(6/198) = 1.17, p>0.3), which was valid for both sleep groups (Sleep Group×Time-on-task×Response Number F(6/198) = 0.97, p>0.4).

In contrast to sleep, practice increased positivity of SPs (Time-on-task: F(1,33) = 4.0, p<0.05). This effect did not differ between cortical regions and the two sleep groups as indexed by analysis of difference SPs (Response Type×ROI: F(7/231) = 2.1, p>0.05; Sleep Group×Response Type×ROI: F(7/231) = 2.39, p>0.05). Accordingly, no significant shifts in topography of SPs with practice was detected (Z-transformed SPs, Time-on-task×Electrode, F(27/891) = 1.1, p>0.2), which was valid for both the Early-NG and Late-NG (Sleep Group×Time-on-task×Electrode, F(27/891) = 0.76, p>0.6) and for both the unpredictable and predictable responses (Sleep Group×Time-on-task×Response Type×Electrode, F(27/891) = 1.8, p>0.05).

### Correlations With Sleep Stages

Correlations were computed between changes in min-max normalized SPs from before to after sleep, separately for the eight ROIs, and the proportion of each sleep stage. Significant correlations were only found for the Early-NG, all of which occurred with SWS, particularly with S4. As shown in Table 2, early-night participants who had more S4 during their sleep did not increase the negative SPs for predictable response processing after sleep (time windows 5, 6, 7), which produced a relative spatial reduction of negativity after sleep (greater difference between pre- and post-sleep min-max normalized SPs) at the left frontal-temporal ROI (LFT) and at parieto-occipital and centro-parietal ROIs (LOP, ROP, and MCP). Also, negative SPs were less focused to the MF region for unpredictable response processing in these subjects, as indicated by correlations of MF with SWS shown in Table 2.

**Table 2:** Significant correlations between proportions of sleep stages and changes of min-max normalized SW across sleep in the eight regions of interest (ROI) for the early-night group (n = 25).

Variables	Pearson r	P
LFT (TWs 5,6) with S4	0.45≤r≤0.50	<0.03
LOP (TWs 5,6,7) with S4	0.40≤r≤0.50	<0.05
ROP (TWs 5,6,7) with S4	0.50≤r≤0.60	<0.05
MCP (TWs 5,6,7) with S4	0.40≤r≤0.50	<0.03
MF (TWs 1,2,3) with S4	0.45≤r≤0.60	<0.04

Most of the ROI-TW combinations that correlated with S4 also correlated with SWS (S3+S4). Being redundant, these SWS results are not compiled.

TW, Time Window; ROIs: LFT, left fronto-temporal, LOP, left occipito-parietal, ROP, right occipito-parietal, MCP, mid centro-parietal, MF, mid-frontal.

## Discussion

The present study quantified slow EEG shifts [36], [38] in order to identify and analyze, at the neurophysiological level of brain functioning, the sleep-related reorganization of neural task representations. One question was if neural reorganization after sleep would depend on the differential pre-activation of hippocampo-cortical networks induced by predictable and unpredictable items in the NRT [30]–[31]. Another objective was to test if implicit conditions of pre-sleep learning could induce alterations in the neurophysiologic mechanisms of the NRT. A final question was if SWS and REM sleep had specific functional roles for the reorganization of neural task representations [19], [39]–[40]. Negative event-related SPs were analyzed to reflect region-specific cortical activations of controlled task processing [34], [36], [38], [40]–[41].

Consistent with the task requirements of the NRT, maximal SP negativities were distributed at left central regions, which reflects the activation of motor cortical areas in relation to movement production with the right hand, at occipital-parietal regions, which reflects intensive processing of visual and visuo-motor associations, and at left fronto-temporal regions, which might reflect the contribution of verbal processing to NRT performance.

One major result was that SPs manifested a regional re-allocation only after early-night but not after late-night sleep, where SPs displayed region-nonspecific and item-nonspecific increases of negativity and thereby basically preserved their pre-sleep scalp distribution. Importantly, the topography shift of SPs after early-night sleep was only expressed for the processing of predictable responses in the second half of the string, which strongly correlated with the amount of SWS. This topography shift was due to a lack of changes in SP magnitude at occipito-parietal regions and a similar lack of changes to predictable responses at left anterior locations accompanied by SP negativization at other electrodes.

The regional re-allocation of negative SPs means that neural networks activated to sub-serve NRT performance after sleep are spatially distinctive from those activated before sleep. Since this re-allocation was basically confined to the processing of those responses that could be predicted by the hidden regularity of the NRT, neural representations of higher-order abstract knowledge of the task appear to be spatially reorganized. However, the abstract structure of the task remained unknown to the subjects both before sleep and in the first block after sleep analyzed here. Obviously, the regional shift of negative SPs for predictable responses may not reflect an activation of a neural system for conscious processing of abstract task regularity. Nor may it reflect mere procedural learning since RT speeding after sleep was similar for unpredictable and predictable responses, and was also similar for the early- and late-night group. Also, sleep-related alterations of attention may not be responsible for the spatial re-allocation of activations since performance variance was not affected by sleep in any of the groups. This finding can be therefore associated with a spatial reorganization of the implicit memory networks, thus providing a neurophysiological evidence for the specific role of SWS, in contrast to REM sleep, for the redistribution of implicit neural representations.

These observations are important in several respects. Firstly, they reveal that the effects of sleep may be covert rather than overt because (a) early- and late-night sleep produced the same behavioral pattern of RT decrease, but changes in electrophysiological parameters were only found after early-night sleep, and (b) post-sleep response speeding did not differ between unpredictable and predictable responses, but neurophysiologic alterations were only found for predictable responses. Traditionally, the lack of sleep-related differences in performance is accepted to index that sleep has no effects. However, covert alterations may turn critically important in directing and refining our understanding about the functional significance of sleep in memory consolidation processes [42].

Secondly, the present results demonstrate that although the pre-sleep learning conditions were implicit, sleep did induce neural alterations. Hence, as previously indicated by Spencer et al. (2006) [13], explicitness at learning may not be the critical determinant of whether memories will be consolidated or not after sleep [21]. More importantly, here neurophysiological evidence is provided that neural task representations were reorganized only for those items in the second half of the string that could be predicted by the hidden structure of the task, the processing of which activates hippocampal and medial temporal lobe more intensively than the processing of unpredictable items [30]–[31]. As reported by Yordanova et al. (2008) [26], in the present data set, RTs were shorter to predictable relative to unpredictable responses in the pre-sleep session, which verifies the acquisition of implicit knowledge before sleep associated with a stronger activation of the hippocampal networks. Of relevance, predictable items did not receive more controlled processing before sleep as evinced by negative SPs. Thus, a preceding stronger pre-activation of hippocampal-cortical networks rather than a different cortical activation by attentional control appears as a critical determinant of off-line restructuring of task representations.

Thirdly, since early and late sleep critically differ in the distribution of SWS and REM sleep (as confirmed by our sleep data here), the present results emphasize the distinct functions of different sleep stages in the process of off-line memory consolidation [2], [19], [28], [40], [43]. In particular, the confinement of electrophysiological indicators of cortical reorganization to early, SWS-dominated sleep, provides support to current understanding that SWS, in contrast to REM sleep, plays an important role in reorganizing the hippocampus-supported memories [5], [15], [18]. Furthermore, the correlations with the amount of SWS support the notion that implicit memory representations are modulated by the deeper sleep stages, as previously concluded from behavioral data [26]. This off-line reorganization of implicit cortical networks may be induced by an enhanced transfer of implicit information as a result of follow-up re-activation of hippocampal-cortical circuits during SWS, since there is a strong activation of the hippocampus when implicit knowledge is accumulated in the course of NRT performance [31], and when the context and relationships between events are processed [44]–[46].

Since early and late nocturnal sleep inherently take place at different times of the day, circadian factors modifying cognitive functioning independent of sleep could have influenced the results. As discussed in Ref. [26], indices of more sleepiness and less activation and concentration were detected in the middle of the night as compared to the evening and the morning although these circadian influences were relatively small compared to activating effects of task performance per se. Thus, retesting in the early-night group and initial practice in the late-night group may have been influenced by somewhat reduced cognitive functioning in the middle of the night. However, the pattern of RT and SP results speaks against a substantial impact of these factors: At initial training, no differences in RT or SPs were found between the two sleep groups, despite the different time-points of practice. Also, even more subjects gained insight into the hidden task structure at retest in the early- than the late-night group, although the retest session in the early- but not in the late-night group took place at the less favourable time-point in the middle of the night. Therefore, diurnal variations in subjective states are unlikely to be a critical factor for current behavioral and SP observations.

Our analyses of slow EEG potentials demonstrated that response speeding after sleep was accompanied by a negativization of SPs, whereas RT decrease as a function of learning with practice was accompanied by a positivization of SPs. This latter observation is consistent with the notion that slow EEG shifts reflect the amount of controlled processing invested to support task-specific regional activations [34], [36], [38], [47] and verifies the automation of NRT processing with practice. Additionally, no specific regional re-allocations of SPs accompanied the time-on-task improvement in performance (Fig. 3A right). With this evidence, it is critical that sleep-related gain in performance is not based on off-line facilitation of automatic processing. Instead, greater resources of cognitive control are allocated for NRT after sleep. Yet, a post-sleep negativization of slow waves did *not* occur at the occipital-parietal regions after early-night sleep, suggesting that no additional controlled processing was needed for the functional activation of these areas, in contrast to late-night sleep. Previous reports have identified the promoting role of sleep for visual-motor learning, although the contribution of different sleep stages has not been specified [1], [40], [48]–[51]. The present results of the relative inactivation of the occipito-parietal regions provide evidence for the supporting effect of SWS to off-line learning within the visual system. Additionally, predictable items could be processed only after early sleep without enhanced activation of cognitive control systems over left fronto-temporal cortices, i.e., in an operationalized mode, which is a clear index for off-line neural facilitation within the implicit memory system [30]. A more general implication of these observations is that they demonstrate that similar changes in behavior may be produced by different neurophysiological processes: RT speeding with practice stemmed from a facilitation of controlled processing due to procedural learning, RT speeding after late night sleep was associated with increase in cognitive effort and controlled task-related activation, and RT speeding after early sleep reflected combined effects of enhanced executive control and implicit learning distributed differentially between task-specific items, unpredictable and predictable. Thus, direct assessment of brain activation patterns during task performance usefully complements behavioral analyses, allowing detection of specific alterations in brain functioning that occur even if no behavioral differences can be observed.

Notably, the presence of sleep-related reorganization of memory representations reflected by slow potentials after early-night sleep did not correlate with the rate of subsequent insight nor was the level of reorganization more expressed in subjects who gained explicit knowledge of hidden task regularities. One explanation might be that reorganization of implicit task representations is not a strict determinant of explicit knowledge generation after sleep. Indeed, a pre-sleep accumulation of implicit knowledge has been shown to be a supporting but not a mandatory condition for gain of insight after sleep, since half of the subjects who discovered NRT structure after sleep had not acquired implicit knowledge of this NRT structure before sleep [26]. It can be therefore suggested that although SWS supports the reorganization of implicit task representations (present results) and their transformation to explicit memories [26], there are additional factors that may critically modulate the probability for subsequent post-sleep insight solutions.

In conclusion, analyses of slow negative potentials as markers of controlled cortical activation provide evidence that slow wave sleep promotes the neural reorganization of implicit task representations and provide neurophysiological evidence for the role of SWS in the consolidation of memories encoded with hippocampo-cortical interaction.

## Materials and Methods

### Ethics Statement

This research was approved by the ethics committee of the University of Lübeck, Lübeck, Germany. Informed written consent was obtained from all subjects prior to the study.

### Subjects

The same sample as in Ref. [26] was used (no task-related EEG results were reported in that paper). Participants were healthy students (18–28 years old) without any history of sleep disturbances or psychiatric or neurological disorders. The total number of subjects of that original sample was 55. Due to technical limitations (low quality or missing EEG records during NRT or sleep) seven subjects were excluded. Twenty-five subjects (7 females) were tested for the first half of the night (early-night group, Early-NG), and 23 subjects (9 females) were tested for the second half of the night (late night group, Late-NG) and were used for statistical comparisons.

All subjects spent an adaptation night in the sleep laboratory including placement of electrodes. Subjects were paid for their participation.

### Task

The task is illustrated in Fig. 1A. The same version of the NRT as described previously in Ref. [28] was used. On each trial, a different string of eight digits was presented, with all digits appearing simultaneously. Each string was composed of the digits 1, 4, and 9. For each string, subjects had to determine a digit defined as the final result of the task trial (Solution). This could be achieved by sequentially processing pairs of digits from left to right according to two simple rules: (1) The “identity rule” states that the result of two identical digits is the same digit (e.g., 4 and 4 gives 4, see Fig. 1A - second Response D). (2) The “difference rule” states that the result of two non-identical digits is the remaining third digit (e.g., 1 and 9 gives 4 - Response A in Fig. 1A; 4 and 1 gives 9 - the next Response B in Fig. 1A).

The 1, 2, and 3 keys on the PC numeric pad were labeled accordingly 1, 4, and 9 and served as response keys. The entered responses appeared on the screen and remained there until the end of the trial, thereby forming a response sequence below the stimulus sequence. To produce the first response, comparisons are made between the first and the second digits from the stimulus string (Fig. 1A–Response A). After processing the first two digits, comparisons are made between this result (appearing in the response string) and the next digit from the stimulus string, then between the result of this new processing and the next digit from the stimulus string, and so on (Fig. 1A). Thus, applying the two rules, subjects generated a string of seven responses, with the last one indicating the final result (Solution) to be confirmed by pressing the “Enter” key on the numeric pad. The time for any single response was limited to 4 s and to a total of 12 s for all responses until pressing ”Enter”. Pressing the ”Enter” key was followed by a change of color of the entered final response on the screen, from red to blue (Fig. 1A – last response B = 9). After another 1-s period, feedback was provided. In case of a correct final result, all digits on the screen, in addition to the final one, changed their color to blue, whereas in case of an incorrect solution, the German word “Wrong” appeared in red on the screen. The screen was cleared after another 0.5 s, and the next trial started.

Instructions stated that only the final result was to be determined for each trial and this could be done at any time. Importantly, unmentioned to the subjects, all strings were generated according to the same underlying regularity, which, if discerned, allowed an early determination of the solution. Specifically, as shown on Fig. 1A – bottom row, all response sequences had the form ABCDDCB (with A, B, C, and D representing one of the digits 1, 4, or 9), such that the last three responses always mirrored the preceding three responses. In this way, the second response in each trial was identical to the final solution. Thus, when gaining insight into this regularity, participants could abruptly shortcut sequential responding by pressing the ”Enter” key already after the second response, whereupon the trial was finished and the next trial started. Note that this regularity is abstract because the actual digit strings and responses changed from trial to trial. Thus, discovery of the rule cannot simply be based on repetition of the same finger movements in all trials.

RTs were measured continuously during task performance, separately for each response in the response string. RT of the first response (R1) was measured as the time from string appearance to the first key press. The RTs of the other responses (R2, R3, R4, R5, R6, R7, Enter) were measured as the time between the previous and the current key press.

### Experimental Design

The experimental design is presented in Fig. 1B. Subjects were tested individually in a sound-attenuated room. They performed a pre-sleep session of initial practice comprising 3 task blocks and a post-sleep retest session of 10 task blocks, with 30 trials in each block. Insight was automatically identified by the program when at least 24 correct short-cuts within the same block occurred, in which case the task was terminated. Initial practice was preceded by extensive standardized instructions given on the computer screen, which included a short training block of 10 task trials. To assure correct understanding of the “identity” and “difference” rule, this block was repeated as long as the subject performed the 10 trials without mistake.

To investigate the effects of different sleep phases, the interval between initial training and retest was filled with three hours of sleep either in the early night, containing high amounts of SWS, or in the late night, containing high amounts of REM sleep (Fig. 1B). In the Early-NG, subjects came to the laboratory at about 21:00 h. After placement of electrodes, they performed the three blocks of initial training (including preceding computer-guided instructions) at about 22:00 h and thereafter went to bed at about 23:00 h. After three hours of sleep in the early night they were awakened to perform the 10 blocks of NRT retesting. Subjects in the Late-NG came to the laboratory at about 22:00 h and, after placement of electrodes, first slept for three hours in the early night (to “consume” SWS) before performing the initial training at about 2:30 h. Then, they slept again for another three-hour period in the late night (about 4:00 h–7:00 h), followed by retesting in the morning. Subjects were only awakened from light sleep stages 1 or 2 to avoid cognitive disturbances that can occur after awakenings from SWS or REM sleep, and were retested 20–30 minutes after awakening to avoid effects of sleep inertia. As an additional control, subjective levels of sleepiness, activation, boredom, concentration, and motivation were assessed on 5-point scales immediately before and after each session of initial training and retest. In all conditions, sessions also included performance in a short simple choice-response task unrelated to the present study, taking place immediately before and after sleep (i.e. after initial NRT training and before NRT retesting).

After NRT retesting, subjects filled in a questionnaire related to their explicit knowledge of the task structure (beginning with open questions, followed by closed questions) as well as possible strategies used during task performance. An additional behavioral test comprised a speeded task in which 15 different strings were presented and subjects had to indicate the final result to each string within 2 s after string presentation. For more details on behavioral and RT measurements, see Ref. [26].

### Sleep EEG and Analysis

Sleep was recorded polysomnographically, including EEG recordings from the left and right central sites (C3, C4), horizontal and vertical EOG, and EMG from chin electrodes. Sleep stages S1, S2, S3, S4, and REM sleep were classified in 30-second epochs according to Ref. [37]. SWS was calculated as the sum of time spent in sleep stages S3 and S4.

### Task EEG Data Acquisition

EEG was recorded continuously during the NRT block performance. EEG recording times are illustrated on Fig. 1B by hatched bars. EEG was recorded with 28 Ag/AgCl scalp electrodes located on the positions AF3, AF4, F7, F3, Fz, F4, F8, FC5, FC1, FC2, FC6, T7, C3, Cz, C4, T8, CP5, CP1, CP2, CP6, P7, P3, Pz, P4, P8, PO3, PO4, and Oz according to the 10-20 International system. The vertical electrooculogram (VEOG) was recorded from electrodes placed above and below the left eye. The horizontal electrooculogram (HEOG) was recorded from electrodes attached to the outer canthi of the eyes. All electrode sites were referenced to linked mastoids. Impedances were maintained below 10 kOhms. EEG and EOG signals were amplified and digitized by using Neuroscan Synamps amplifiers, with pass-band filter of 0.03–70 Hz, and sampling frequency of 250 Hz.

### Analysis of Slow Potentials

Data processing was performed with Brain Vision Analyzer version 1.05 (Brain Products GmbH, Gilching, Germany). The EEG was segmented into epochs of 10 s duration, from 0.5 s before to 9.5 s after appearance of the stimulus digit string. EEG traces were visually inspected for gross EOG and EMG artifacts. Contaminated trials were discarded. Slight horizontal and vertical eye movements preserved in the accepted trials were corrected by means of a linear regression method for EOG correction [52].

These epochs were averaged across trials, separately for the pre-sleep and post-sleep sessions. For the pre-sleep session, the last 35 artifact-free trials (most of which were from the last block 3) were used, and for the post-sleep session, the first 35 artifact-free trials (most of which were from the first of the 10 retest blocks) were used (Fig. 1B, blue shading on the hatched bars). To evaluate time-on-task effects, 35 artifact-free trials were selected from the very end of the retest post-sleep session (most of them were from the last tenth block) and were compared with trials from the beginning of the pre-sleep session. For each subject, block, and electrode, mean values were measured for 8 consecutive 1-s time windows starting 1.5 s after string onset (Fig. 2B). This starting point was chosen to avoid stimulus-related phenomena such as P300 or other slow ERP components.

To perform topography analyses, two types of normalization were applied to the data, z-score transformation and min-max normalization. By means of z-scoring, the mean value of all electrode measurements was subtracted from the measured value of each single electrode which was then divided by the standard deviation of the same measurements. In such a way, in the z-scored values, data variance was taken into account twice – by means of first order (mean value) and second order (SD) statistics [34]. The analysis design for z-transformed SPs was Session (pre-sleep vs. post-sleep, as defined above)×Response Type×Electrode (28 electrodes). Repeated measures ANOVAs were carried out separately for the Early-NG and Late-NG. The major focus of this analysis was on Session×Electrode and Session×Response Type×Electrode interactions. Min-max normalization was performed additionally to test the topography distribution. It was calculated by taking minimal-to-maximal values from all electrode measurements as 100% (for similar procedures, see Ref. [53]).

For repeated-measures variables with more than two levels, the Greenhouse-Geisser correction procedure was employed, with original degrees of freedom and corrected probabilities (P) being reported.

## Supporting Information

Text S1 Supplemental information concerning the different effects of early- and late-night sleep on the spatial reorganization of slow negative potentials for unpredictable and predictable responses in the number reduction task.
(0.04 MB DOC)

Figure S1 Grand average event-related slow potentials (SPs) for the early- (Early-NG) and late-night group (Late-NG). Time dynamics of group mean values for three regions of interest (ROIs: left fronto-temporal, LFT; left central, LC; left occipito-parietal, LOP) is presented at the two most-left panels. Amplitudes are min-max normalized and presented as percentages. Time windows are labeled 1 to 8 as presented in Fig. 2. Topography distribution of SPs from both groups for the unpredictable and predictable response types is presented as difference maps (post-sleep, POST minus pre-sleep, PRE) at the two most-right panels.
(2.50 MB TIF)

## References

[1] Karni A, Tanne D, Rubenstein BS, Askenasy JJ, Sagi D (1994). Dependence on REM sleep of overnight improvement of a perceptual skill.. Science.

[2] Maquet P (2001). The role of sleep in learning and memory.. Science.

[3] Walker MP, Brakefield T, Morgan A, Hobson JA, Stickgold R (2002). Practice with sleep makes perfect: Sleep-dependent motor skill learning.. Neuron.

[4] Walker MP, Stickgold R (2004). Sleep-dependent learning and memory consolidation.. Neuron.

[5] Born J, Rasch B, Gais S (2006). Sleep to remember.. Neuroscientist.

[6] Robertson EM (2009). From creation to consolidation: A novel framework for memory processing.. PLoS Biol.

[7] Marshall L, Born J (2007). The contribution of sleep to hippocampus-dependent memory consolidation.. Trends Cogn Sci.

[8] Gais S, Albouy G, Boly M, Dang-Vu TT, Darsaud A (2007). Sleep transforms the cerebral trace of declarative memories.. Proc Natl Acad Sci U S A.

[9] Schabus M, Gruber G, Parapatics S, Sauter C, Klösch G (2004). Sleep spindles and their significance for declarative memory consolidation.. Sleep.

[10] Squire LR (1992). Memory and the hippocampus: a synthesis from findings with rats, monkeys, and humans.. Psychol Rev.

[11] Forkstam C, Petersson KM (2005). Towards an explicit account of implicit learning.. Curr Opin Neurol.

[12] Graves LA, Heller EA, Pack AI, Abel T (2003). Sleep deprivation selectively impairs memory consolidation for contextual fear conditioning.. Learn Mem.

[13] Spencer RM, Sunm M, Ivry RB (2006). Sleep-dependent consolidation of contextual learning.. Curr Biol.

[14] Rasch B, Büchel C, Gais S, Born J (2007). Odor cues during slow-wave sleep prompt declarative memory consolidation.. Science.

[15] Rasch B, Born J (2007). Maintaining memories by reactivation.. Curr Opin Neurobiol.

[16] Tse D, Langston RF, Kakeyama M, Bethus I, Spooner PA (2007). Schemas and memory consolidation.. Science.

[17] Yeshenko O, Mölle M, Marshall L, Born J, Sara SJ (2006). Locus coeruleus firing during SWS is time-locked to slow oscillations: possible contribution of the noradrenergic system to off-line information processing in rats.. J Sleep Res.

[18] Marshall L, Helgadóttir H, Mölle M, Born J (2006). Boosting slow oscillations during sleep potentiates memory.. Nature.

[19] Plihal W, Born J (1999). Effects of early and late nocturnal sleep on priming and spatial memory.. Psychophysiology.

[20] Reber PJ, Squire LR (1994). Parallel brain systems for learning with and without awareness.. Learn Mem.

[21] Robertson EM, Pascual-Leone A, Press DZ (2004). Awareness modifies the skill-learning benefits of sleep.. Curr Biol.

[22] Degonda N, Mondadori CR, Bosshardt S, Schmidt CF, Boesiger P, Nitsch RM, Hock C, Henke K (2005). Implicit associative learning engages the hippocampus and interacts with explicit associative learning.. Neuron.

[23] Greene AJ (2007). Human hippocampal-dependent tasks: is awareness necessary or sufficient?. Hippocampus.

[24] Woltz DJ, Bell BG, Kyllonen PC, Gardner MK (1996). Memory for order of operations in the acquisition and transfer of sequential cognitive skills.. J Exp Psych Learn Mem Cogn.

[25] Frensch PA, Haider H, Rünger D, Neugebauer U, Voigt S, Jimenez L (2002). Verbal report of incidentally experienced environmental regularity: The route from implicit learning to verbal expression of what has been learned.. Attention and implicit learning.

[26] Yordanova, Kolev V, Verleger R, Bataghva Z, Born J (2008). Different roles of early and late night sleep in making implicit knowledge explicit.. Learn Mem.

[27] Lang S, Kanngieser N, Jaskowski P, Haider H, Rose M (2006). Precursors of insight in event-related brain potentials.. J Cogn Neurosci.

[28] Wagner U, Gais S, Haider H, Verleger R, Born J (2004). Sleep inspires insight.. Nature.

[29] Haider H, Rose M (2007). How to investigate insight: A proposal.. Methods.

[30] Rose M, Haider H, Weiller C, Büchel C (2002). The role of medial temporal lobe structures in implicit learning: an event-related fMRI study.. Neuron.

[31] Rose M, Haider H, Büchel C (2005). Unconscious detection of implicit expectancies.. J Cogn Neurosci.

[32] Rose M, Haider H, Weiller C, Büchel C (2004). The relevance of the nature of learned associations for the differentiation of human memory systems.. Learn Mem.

[33] Ruchkin DS, Picton TW (1988). Measurement of event-related potentials: signal extraction.. Event-related potentials. EEG Handbook, rev. series, vol. 3.

[34] Rösler F, Heil M, Henninghausen E (1995). Distinct cortical activation patterns during long-term memory retrieval of verbal, spatial and color information.. J Cogn Neurosci.

[35] Khader P, Burke M, Bien S, Ranganath C, Rösler F (2005). Content-specific activation during associative long-term memory retrieval.. NeuroImage.

[36] Rösler F, Heil M, Röder B (1997). Slow negative brain potentials as reflections of specific modular resources of cognition.. Biol Psychol.

[37] Rechtschaffen A, Kales AA (1968). Manual of standardized terminology, techniques and scoring system for sleep stages of human subjects.

[38] Khader P, Knoth K, Burke M, Ranganath C, Bien S (2007). Topography and dynamics of associative long-term memory retrieval in humans.. J Cogn Neurosci.

[39] Fischer S, Drosopoulos S, Tsen J, Born J (2006). Implicit learning - explicit knowing: a role for sleep in memory system interaction.. J Cogn Neurosci.

[40] Gais S, Born J (2004). Declarative memory consolidation: Mechanisms acting during human sleep.. Learn Mem.

[41] Rockstroh B, Elbert T, Canavan A, Lutzenberger W, Birbaumer N (1989). Slow cortical potentials and behaviour.

[42] Orban P, Rauchs G, Balteau E, Degueldre C, Luxen A (2006). Sleep after spatial learning promotes covert reorganization of brain activity.. Proc Natl Acad Sci U S A.

[43] Drosopoulos S, Wagner U, Born J (2005). Sleep enhances explicit recollection in recognition memory.. Learn Mem.

[44] Cohen NJ, Ryan J, Hunt C, Romine L, Wszalek T (1999). Hippocampal system and declarative relational. memory: summarizing the data from functional neuroimaging studies.. Hippocampus.

[45] Henke K, Weber B, Kneifel S, Wieser HG, Buck A (1999). Human hippocampus associates information in memory.. Proc Natl Acad Sci U S A.

[46] Elsner B, Hommel B, Mentschel C, Drzezga A, Prinz W (2002). Linking actions and their perceivable consequences in the human brain.. NeuroImage.

[47] Heil M, Rösler F, Hennighausen E (1997). Topography of brain electrical activity dissociates the retrieval of spatial versus verbal information from episodic long-term memory in humans.. Neurosci Lett.

[48] Verleger R, Schuknecht SV, Jaśkowski P, Wagner U (2008). Changes in processing of masked stimuli across early- and late-night sleep: a study on behavior and brain potentials.. Brain Cogn.

[49] Stickgold R, James L, Hobson JA (2000). Visual discrimination learning requires sleep after training.. Nat Neurosci.

[50] Stickgold R (2005). Sleep-dependent memory consolidation.. Nature.

[51] Gais S, Plihal W, Wagner U, Born J (2000). Early sleep triggers memory for early visual discrimination skills.. Nat Neurosci.

[52] Gratton G, Coles MGH, Donchin E (1983). A new method for off-line removal of ocular artifact.. Electroencephalogr Clin Neurophysiol.

[53] McCarthy G, Wood C (1985). Scalp distributions of event-related potentials: an ambiguity associated with analysis of variance models.. Electroencephalogr Clin Neurophysiol.

