# Supplementary material for: Covert Reorganization of Implicit Task Representations by Slow Wave Sleep
Source: PLoS One. 2009 May 25;4(5):e5675. doi: 10.1371/journal.pone.0005675 (PMC2682605; doi:10.1371/journal.pone.0005675)
Supplement: Text S1 — Supplemental information concerning the different effects of early- and late-night sleep on the spatial reorganization of slow negative potentials for unpredictable and predictable responses in the number reduction task. (0.04 MB DOC) [file pone.0005675.s001.doc]

**Supporting Information**

To characterize the changes of SP topography from the session before sleep to after sleep independently of individual variations in SP magnitude min-max normalized values of SPs were analyzed. Analyses were done separately for the left and right-hemisphere ROIs, because of the asymmetric SP distribution, and also because major differences between the effects of early and late sleep on SPs were found over the left-hemisphere. Figure S1 demonstrates sleep-related spatial re-distribution of left-hemisphere SPs. Before sleep, the two sleep groups had similar relations among left ROIs (LFT, LC, LOP) in the course of string processing (Sleep Group x ROI and Sleep Group x ROI x Response Type, p > 0.7). The pre-sleep topography pattern was characterized by a specific U-shaped dynamics of the negative SP at LFT, and comparable negativities at LC and LOP (around 35 %), slightly decreasing in the course of string processing. After late sleep, this same pattern was basically preserved (Session x ROI x Response Type in the Late-NG group, F(2/44) = 0.14, p > 0.7). Yet, after early sleep, this pattern changed (Session x ROI x Response Type in the Early-NG: F(2/48) = 4.93, p = 0.01). This was due to the relative deactivation of the LOP ROI (effect of Session for this ROI in the Early-NG: F(1/24) = 3.2, p < 0.05, Fig. 4), which produced a remarkable dissociation between SP activity at the LC and the LOP mainly in the period of unpredictable response processing (LC vs. LOP for unpredictable responses: F(1/24) = 5. 35, p < 0.05), in contrast to the pre-sleep session. Also, the lack of post-sleep negativization of SPs for predictable responses at left anterior sites after early sleep produced not only a dissociation between LOP and LC but also similar SP negativities at LFT and LC for predictable responses (ROI x Response type for LFT and LC: F(1/24) = 1.57, p > 0.2; LFT vs. LC, p > 0.8).

No differential effects were obtained in analyses of right-hemisphere ROIs: Sleep Group x Session x ROI (F(2/92) = 0.05, p > 0.9) and Sleep Group x Session x Response Type x ROI (F(2/92) = 0.9, p > 0.5), indicating that the regional pattern of right hemisphere ROIs did not change across sleep groups and sleep conditions. Likewise, no differential effects of early vs. late sleep were detected for the mid-line centro-parietal ROI MCP and the mid-frontal ROI MF.
