# Supplementary figures and images for: Covert Reorganization of Implicit Task Representations by Slow Wave Sleep
Source: PLoS One. 2009 May 25;4(5):e5675. doi: 10.1371/journal.pone.0005675 (PMC2682605; doi:10.1371/journal.pone.0005675)

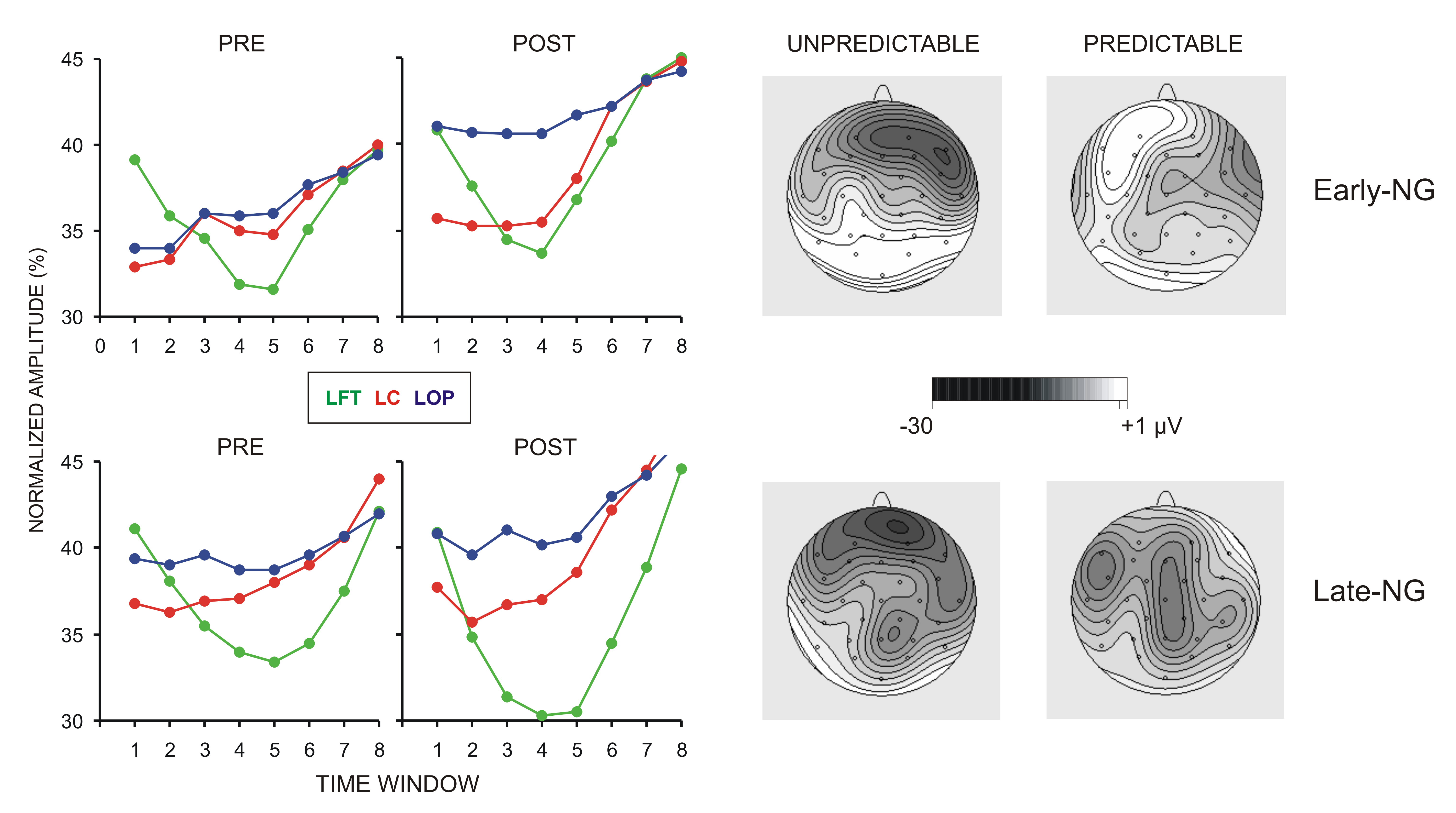

Supplement: Figure S1 — Grand average event-related slow potentials (SPs) for the early- (Early-NG) and late-night group (Late-NG). Time dynamics of group mean values for three regions of interest (ROIs: left fronto-temporal, LFT; left central, LC; left occipito-parietal, LOP) is presented at the two most-left panels. Amplitudes are min-max normalized and presented as percentages. Time windows are labeled 1 to 8 as presented in Fig. 2. Topography distribution of SPs from both groups for the unpredictable and predictable response types is presented as difference maps (post-sleep, POST minus pre-sleep, PRE) at the two most-right panels. (2.50 MB TIF) [file pone.0005675.s002.tif]
